# Anti-Fibrotic Effect of Synthetic Noncoding Oligodeoxynucleotide for Inhibiting mTOR and STAT3 via the Regulation of Autophagy in an Animal Model of Renal Injury

**DOI:** 10.3390/molecules27030766

**Published:** 2022-01-25

**Authors:** Hyun Jin Jung, Hyun-Jin An, Mi-Gyeong Gwon, Hyemin Gu, Seongjae Bae, Sun-Jae Lee, Young-Ah Kim, Jaechan Leem, Kwan-Kyu Park

**Affiliations:** 1 Department of Urology, College of Medicine, Catholic University of Daegu, Daegu 42472, Korea; hnjini@cu.ac.kr; 2 Department of Pathology, College of Medicine, Catholic University of Daegu, Daegu 42472, Korea; ahj119@cu.ac.kr (H.-J.A.); daldy88@cu.ac.kr (M.-G.G.); guhyemin07@cu.ac.kr (H.G.); zz22@cu.ac.kr (S.B.); pathosjlee@cu.ac.kr (S.-J.L.); youngah7840@naver.com (Y.-A.K.); 3 Department of Immunology, College of Medicine, Catholic University of Daegu, Daegu 42472, Korea; jcim@cu.ac.kr

**Keywords:** renal fibrosis, autophagy, oligodeoxynucleotide, decoy, antisense, mTOR, STAT3

## Abstract

Renal fibrosis is a common process of various kidney diseases. Autophagy is an important cell biology process to maintain cellular homeostasis. In addition, autophagy is involved in the pathogenesis of various renal disease, including acute kidney injury, glomerular diseases, and renal fibrosis. However, the functional role of autophagy in renal fibrosis remains poorly unclear. The mammalian target of rapamycin (mTOR) plays a negative regulatory role in autophagy. Signal transducer and activator of transcription 3 (STAT3) is an important intracellular signaling that may regulate a variety of inflammatory responses. In addition, STAT3 regulates autophagy in various cell types. Thus, we synthesized the mTOR/STAT3 oligodeoxynucleotide (ODN) to regulate the autophagy. The aim of this study was to investigate the beneficial effect of mTOR/STAT3 ODN via the regulation of autophagy appearance on unilateral ureteral obstruction (UUO)-induced renal fibrosis. This study showed that UUO induced inflammation, tubular atrophy, and tubular interstitial fibrosis. However, mTOR/STAT3 ODN suppressed UUO-induced renal fibrosis and inflammation. The autophagy markers have no statistically significant relation, whereas mTOR/STAT3 ODN suppressed the apoptosis in tubular cells. These results suggest the possibility of mTOR/STAT3 ODN for preventing renal fibrosis. However, the role of mTOR/STAT3 ODN on autophagy regulation needs to be further investigated.

## 1. Introduction

A study has reported that 10% of the world’s population suffers from chronic kidney disease (CKD) [1]. Regardless of their cause, most forms of CKD are characterized by inflammation and progressive fibrosis [2]. Once renal fibrosis develops, most CKD patients will progress to an irreversible end-stage renal disease, in which kidney transplantation with dialysis is the only therapeutic option.

Renal fibrosis is a pathophysiological process that is characterized by tubulointerstitial fibrosis (TIF) and glomerulosclerosis, and it is the final outcome of various renal diseases [3]. Many cellular and molecular events occur in renal fibrosis, such as the activation of interstitial myofibroblasts, epithelial–mesenchymal transition (EMT) and/or endothelial–mesenchymal transition, extracellular matrix (ECM) deposition, microvascular dysfunction, and autophagy [4,5]. Renal fibrosis is initiated and sustained by many prosclerotic factors, including transforming growth factor (TGF)-β1, which can not only increase the expression of matrix proteins but also induce EMT in renal cells [6]. TGF-β1 is an essential regulator of ECM synthesis and cell proliferation, and it is considered a marker of renal fibrogenesis. In addition, it is noteworthy that the function of autophagy is one of the causes of renal fibrosis after injury [7].

Autophagy, a genetically controlled pathway, is an important cellular mechanism for intracellular lysosome-mediated degradation of damaged organelles, protein aggregates, and other macromolecules in the cytoplasm, and it regulates cell death under normal physiological and pathological conditions [8,9]. Autophagy is also involved in renal diseases, including acute kidney injury (AKI), glomerular diseases, and TIF [10,11,12]. Autophagy deficiency leads to the accumulation of intracellular metabolic wastes, and it has been implicated in various diseases including CKD, neurodegeneration, aging, infectious disease, inflammation, and cancer [13,14,15]. The appropriate enhancement of autophagic activity removes damaged organelles, reduces the intracellular accumulation of abnormal proteins, and promotes pathogen clearance and thereby contributes to cell survival. However, excessive autophagy may induce cell senescence and apoptosis [16]. Autophagy dysfunction is often associated with fibrotic kidney diseases [17,18]. However, the role of autophagy in TIF is complex and inconsistent. Autophagy seems to negatively regulate TGF-β1 signaling, partly through the degradation of TGF-β1 [19] and collagen I [20]. In this study, the relationship between the change in autophagy and renal fibrosis after injury was evaluated.

The mammalian target of rapamycin (mTOR) signaling pathway has been established to be involved in cellular growth, metabolism, and the negative regulation of autophagy [21]. mTOR is an important serine/threonine protein kinase that plays a negative regulatory role in autophagy. mTOR activation can inhibit autophagosome formation [22]. mTOR expression is affected by the signal transducer and activator of transcription (STAT) 3-mediated regulation of autophagy [16]. STAT3 has been reported to be a transcriptional activator of Bcl-2 [23,24]. It moves into the nucleus to activate Bcl-2 and induces its expression [25,26]. The downregulation of STAT3 and Bcl-2 expression can induce autophagy [27]. A previous study [16] showed that the inhibition of oxidative stress and interference with mTOR/STAT3 activity suppressed the autophagy level to a certain degree, and this was conducive to delaying cell senescence [28]. This study evaluated how the change in autophagy appearance by the inhibition of mTOR/STAT3 function affects renal fibrosis after injury. In addition, decoy oligodeoxynucleotide (ODN) was synthesized to control the function of autophagy. A unilateral ureteral obstruction (UUO) mouse model was used to induce a change in autophagy and fibrosis in experiments. To determine the role of autophagy in renal fibrosis, many studies have used the UUO model [29]; this model exhibits the induction of autophagy accompanied by tubular atrophy and interstitial fibrosis [11,30].

The decoy ODN strategy used in this study blocks the transcription factors of a specific gene that can recognize their consensus binding sequences. Previous studies [31,32] demonstrated that the effects of decoy ODNs significantly regulate transcription factors of several disorders. Lee et al. [33] reported the efficacy of synthetic decoy ODNs using NF-kB and Sp1 in an animal model of atherosclerosis. Yuan et al. [34] reported that dual AP-1 and Smad decoy ODN inhibited fibrosis associated with acute dermal wounds in mice through the inhibition of proinflammatory and anti-fibrotic effects.

It remains unclear whether autophagy inhibition has a therapeutic effect on renal injury. Thus, it is necessary to examine the therapeutic effect of autophagy inhibition and the underlying mechanism in an animal model of renal injury. This study investigates the role of autophagy in renal inflammation and the underlying potential molecular mechanisms. It also clarifies the mechanism of mTOR and STAT3 functions for autophagy. Toward these ends, it investigates the inhibitory effects of mTOR/STAT3 decoy ODN on preventing renal fibrosis. The mTOR/STAT3 decoy ODN was designed to inhibit both mRNA expressions of mTOR and STAT3 transcription factors in the UUO kidney mouse model.

## 2. Results

### 2.1. Construction of mTOR/STAT3 Synthetic ODNs

**Figure 1:**
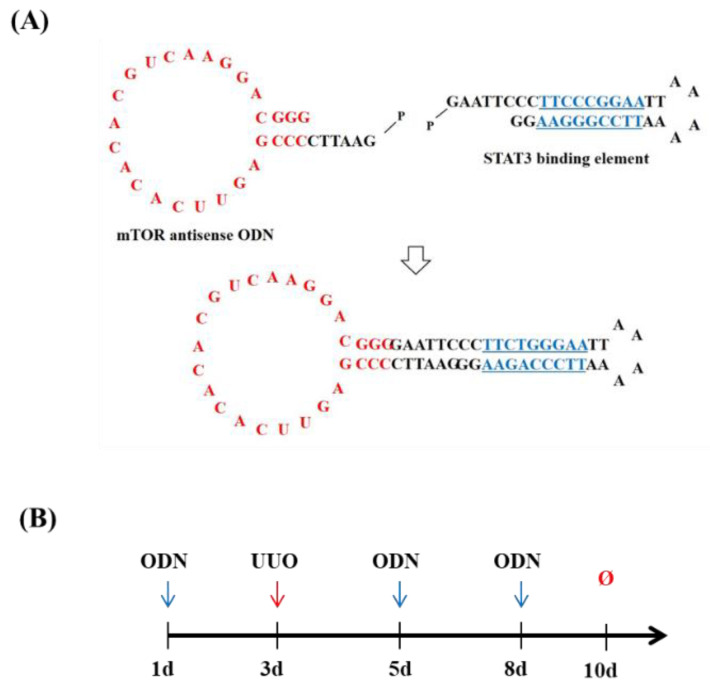
Construction of mTOR/STAT3 Synthetic ODNs. (**A**) Structure of mTOR/STAT3 synthetic ODNs. (**B**) Animal model and transfection of ODN experiment design.

We first designed the mTOR/STAT3 synthetic ODN. The target sites for mTOR mRNA for mTOR antisense ODN were selected via the sequential overlap simulation of secondary structures using the S-Fold program. The STAT3 decoy ODN of ring-type and with a double strand was synthesized to stabilize the structure from the nuclease (Figure 1A). To inhibit the action of mTOR and STAT3, we initially designed synthetic dual-function mTOR/STAT3 synthetic ODN containing a specific complimentary sequence of mTOR mRNA and the consensus sequence of STAT3 transcription factor. To confirm the beneficial effect of mTOR/STAT3 synthetic ODN in renal fibrosis, this study used a UUO-induced obstructive mouse model. The experimental procedure of UUO surgery and ODN transfection is described schematically in Figure 1B.

### 2.2. mTOR/STAT3 Synthetic ODN Attenuated Morphological Change and Improved Kidney Function in UUO Kidney

**Figure 2:**
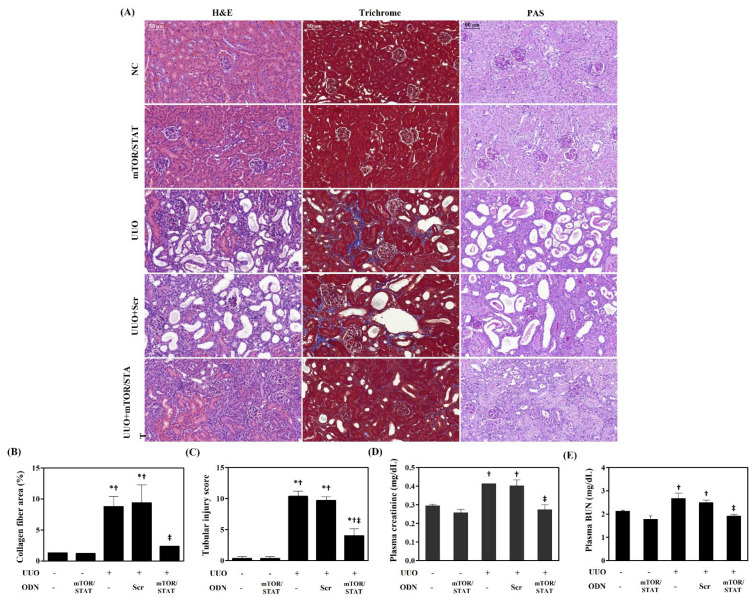
The effect of mTOR/STAT3 ODN on kidney failure and histological change in UUO mice. (**A**) Histopathological alterations in slides stained with H&E, Masson’s trichrome, and PAS. These are representative images from each study group. Scale bar = 50 μm. (**B**) The quantitative analysis of blue-stained collagen in trichrome staining. (**C**) Tubular injury was semiquantitatively scored using PAS-stained sections. (**D**) Serum creatinine and (**E**) BUN concentrations were measured to assess renal function. NC, normal control; mTOR/STAT, normal mice injected with mTOR/STAT3 synthetic ODNs; UUO, UUO surgery; UUO+Scr, underwent UUO surgery and were injected with scrambled ODNs; and UUO+mTOR/STAT, underwent UUO surgery and were injected with mTOR/STAT3 synthetic ODNs. * *p* < 0.05 vs. NC group. † *p* < 0.05 vs. mTOR/STAT group. ‡ *p* < 0.05 vs. UUO group.

The effects of mTOR/STAT3 synthetic ODN on the morphological change caused by the UUO mouse model were investigated. As shown in Figure 2A, no significant morphological change was seen in the glomeruli and tubules in the NC and mTOR/STAT groups. In the UUO and UUO+Scr groups, inflammatory cell infiltration, renal tubular dilatation, atrophy or necrosis, and interstitial fibrosis can be detected easily. However, mTOR/STAT3 synthetic ODN administration markedly reduced these changes in the UUO+mTOR/STAT group. To observe collagen deposition and renal fibrosis, Masson’s trichrome staining was performed. It showed that collagen fiber was deposited in the renal tubules, and renal interstitial fibrosis could be detected in the UUO group. However, mTOR/STAT3 synthetic ODN treatment significantly reduced the renal interstitial fibrosis. In addition, PAS staining was used to assess the degree of damaged renal tubules. PAS staining has been shown to result in higher interstitial thickening and decreased cytoplasm and to induce significant interstitial damage in UUO kidneys compared to the NC and mTOR/STAT groups. These changes were reduced by synthetic mTOR/STAT3 ODN treatment.

The serum levels of serum creatinine and BUN are the classical indicators of renal function. In this study, the serum creatinine and BUN level were examined after UUO surgery (Figure 2B). The serum creatinine and BUN concentrations were increased in the UUO and UUO+Scr groups; this was consistent with previous studies, and it indicated the deterioration of renal function. However, mTOR/STAT3 synthetic ODN treatment markedly reduced the UUO-induced serum creatinine and BUN levels. These results indicated that mTOR/STAT3 ODN treatment alleviated UUO-induced renal injury and improved renal function.

### 2.3. mTOR/STAT3 Synthetic ODN Attenuates UUO-Induced Kidney Tubular Injury

**Figure 3:**
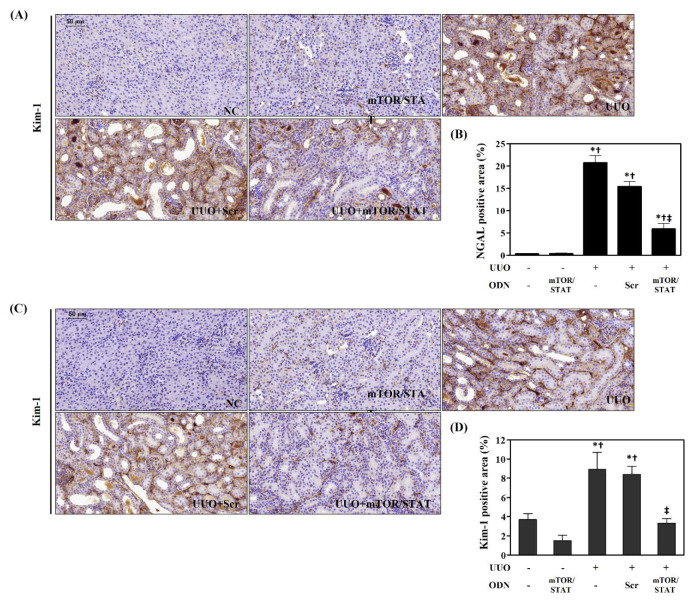
mTOR/STAT3 synthetic ODN alleviates UUO-induced kidney injury. (**A**) Histological images of IHC of NGAL and (**B**) graphs indicating the relative percentage of NGAL expression. These are representative images from each study group. (**C**) Histological images of IHC of Kim-1 and (**D**) graphs indicating the relative percentage of Kim-1 expression. These are representative images from each study group. Scale bar = 50 μm. NC, normal control; mTOR/STAT, normal mice injected with mTOR/STAT3 synthetic ODNs; UUO, UUO surgery; UUO+Scr, underwent UUO surgery and were injected with scrambled ODNs; and UUO+mTOR/STAT, underwent UUO surgery and were injected with mTOR/STAT3 synthetic ODNs. * *p* < 0.05 vs. NC group. † *p* < 0.05 vs. mTOR/STAT group. ‡ *p* < 0.05 vs. UUO group.

To investigate the effects of mTOR/STAT3 synthetic ODN on UUO-induced tubular injury, IHC staining was performed to observe the expression of NGAL and Kim-1, both of which are biomarkers of tubular injury. UUO surgery resulted in significantly increased NGAL deposition in the distal tubules and glomerular, which could be suppressed by mTOR/STAT3 ODN administration (Figure 3A). In addition, the expression of Kim-1 in renal tissues was evaluated by IHC staining. As shown in Figure 3B, Kim-1 expression markedly increased in the UUO and UUO+Scr groups. However, mTOR/STAT3 treatment effectively inhibited UUO-induced Kim-1 expression. These findings suggest that mTOR/STAT3 ODN mitigated UUO-induced kidney damage in mice.

### 2.4. mTOR/STAT3 Synthetic ODN Inhibited UUO-Induced Kidney Inflammation and Immune Cell Infiltration

**Figure 4:**
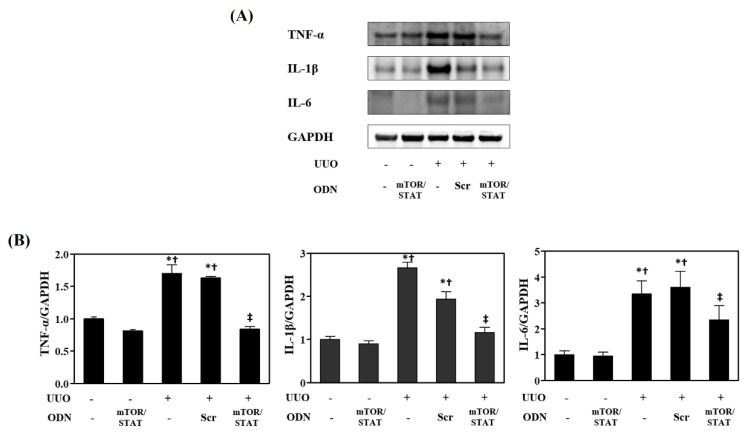
mTOR/STAT3 synthetic ODN suppresses renal inflammation in UUO mice. (**A**) Western blot results showing inflammatory cytokines expression in kidney tissue; (**B**) The graph summarizes the quantification of molecules, each normalized to GAPDH. * *p* < 0.05 vs. NC group. † *p* < 0.05 vs. mTOR/STAT group. ‡ *p* < 0.05 vs. UUO group.

The effects of mTOR/STAT3 synthetic ODN on the production of the inflammatory cytokines TNF-α, IL-1β, and IL-6 was examined to study the anti-inflammatory effect of mTOR/STAT3 ODN on UUO-induced CKD. As shown in Figure 4, the production of TNF-α, IL-1β, and IL-6 was significantly increased in the UUO and UUO+Scr groups compared with the NC and mTOR/STAT groups. However, mTOR/STAT3 synthetic ODN inhibited UUO-induced TNF-α, IL-1β, and IL-6 expression.

**Figure 5:**
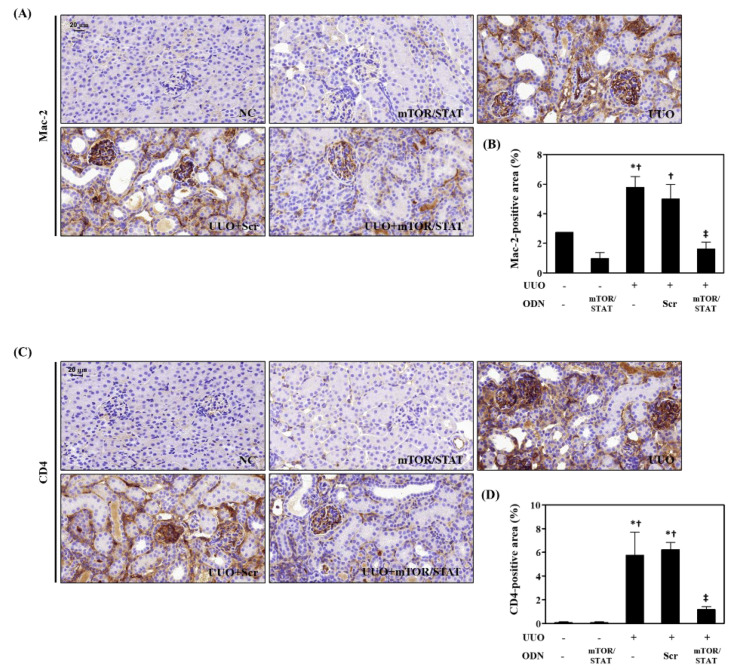
Effect of mTOR/STAT3 synthetic ODN on inflammatory cells infiltration in UUO-injured kidney. (**A**) Histological images of IHC of Mac-2 and (**B**) graphs indicating the relative percentage of Mac-2 expression. These are representative images from each study group. (**C**) Histological images of IHC of CD4 and (**D**) graphs indicating the relative per-centage of CD4 expression. These are representative images from each study group. Scale bar = 20 μm. NC, normal control; mTOR/STAT, normal mice injected with mTOR/STAT3 synthetic ODNs; UUO, UUO surgery; UUO+Scr, underwent UUO surgery and were injected with scrambled ODNs; and UUO+mTOR/STAT, underwent UUO surgery and were injected with mTOR/STAT3 synthetic ODNs. * *p* < 0.05 vs. NC group. † *p* < 0.05 vs. mTOR/STAT group. ‡ *p* < 0.05 vs. UUO group.

The levels of Mac-2 and CD4 expression in kidney tissue were examined after UUO surgery. Mac-2 was expressed on the surface of inflammatory macrophages and several macrophage cells. In the UUO group, as the kidney injury progressed, Mac-2 expression was upregulated in tubular cells and interstitial cells. In contrast, Mac-2 expression was significantly reduced in the UUO+mTOR/STAT group (Figure 5A). IHC staining indicated that CD4 immune cell infiltration was increased after UUO compared with that in the NC and mTOR/STAT groups. However, mTOR/STAT3 ODN treatment significantly reduced immune cell infiltration after obstructive injury (Figure 5B). Collectively, these results indicate that mTOR/STAT3 synthetic ODN plays a role in preventing immune cell infiltration.

### 2.5. mTOR/STAT3 Synthetic ODN Attenuates UUO-Induced Kidney Damage

**Figure 6:**
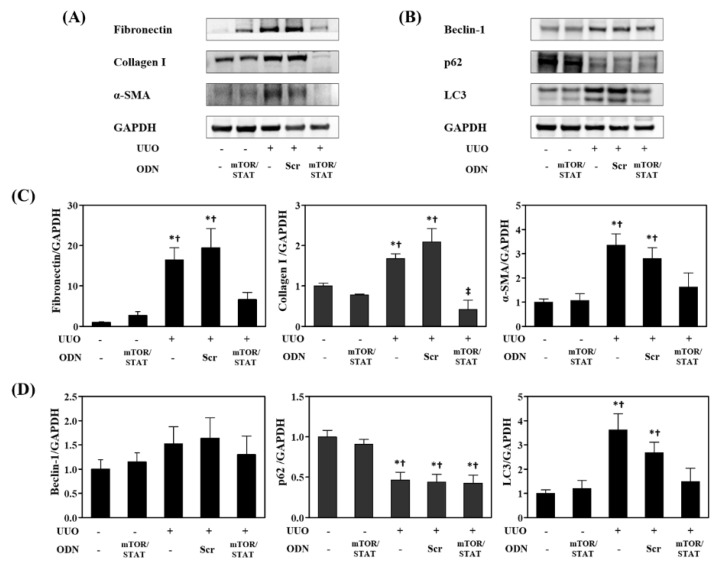
Effects of mTOR/STAT3 synthetic ODN on renal fibrosis and autophagy. (**A**) Western blot results showed that mTOR/STAT ODN attenuated the expression of fibronectin, collagen I, and α-SMA in UUO mice; (**B**) Western blot results of beclin-1, p62, LC3, and GAPDH; (**C**,**D**) The graphs summarize the quantification of molecules, each normalized to GAPDH. * *p* < 0.05 vs. NC group. † *p* < 0.05 vs. mTOR/STAT group. ‡ *p* < 0.05 vs. UUO group.

To evaluate whether mTOR/STAT3 synthetic ODN treatment could affect the levels of ECM and fibrosis markers, we performed Western blotting on the kidney tissue (Figure 6A,C). The protein levels of fibronectin and collagen I were markedly increased in both the UUO and UUO+Scr groups. However, these increases were significantly attenuated by mTOR/STAT3 ODN. In addition, the Western blots revealed that the levels of α-smooth muscle actin (α-SMA), a specific marker for myofibroblast activation, were significantly increased in UUO kidneys. The expression of α-SMA was decreased by mTOR/STAT3 ODN.

Autophagy and apoptosis are two interconnected pathways in response to cellular stress. To investigate whether the kidney protective effect of mTOR/STAT3 ODN was associated with autophagy, the protein expression levels of the autophagy markers Beclin-1, p62, and LC3 were analyzed using Western blots (Figure 6B,D). Beclin-1 and LC3 expression were increased in the UUO group compared to the NC and mTOR/STAT groups. However, mTOR/STAT3 ODN treatment decreased Beclin-1 and LC3 expression nonsignificantly. p62, a substrate of autophagy, incorporates into autophagosomes through direct binding to LC3, and it is efficiently degraded by autophagy. The total expression level of p62 is inversely correlated with autophagic activity [35]. In this study, the protein expression level of p62 was markedly reduced in UUO mice. These results confirmed that mTOR/STAT3 ODN has a renal protective effect. However, there was no statistically significant relation between mTOR/STAT3 ODN treatment and autophagy expression.

### 2.6. mTOR/STAT3 Synthetic ODN Inhibited UUO-Induced Tubular Cell Apoptosis

**Figure 7:**
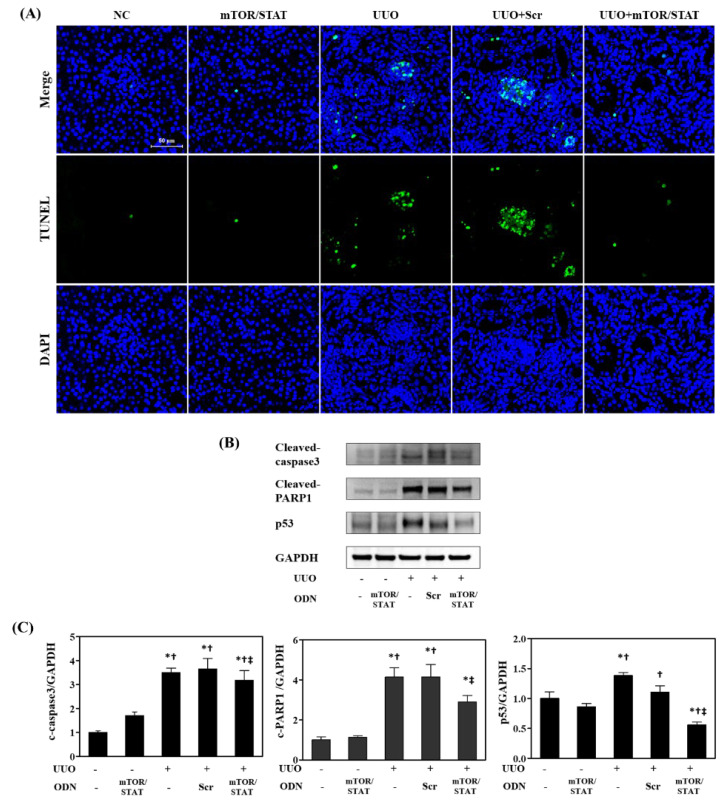
mTOR/STAT3 synthetic ODN inhibited UUO-induced tubular cell apoptosis. (**A**) TUNEL staining in kidneys; (**B**) Western blot results of cleaved caspase-3, cleaved poly (ADP-ribose) polymerase-1 (PARP-1), p53, and GAPDH. These are representative images from each study group. Scale bar = 50 μm. NC, normal control; mTOR/STAT, injected with mTOR/STAT3 synthetic ODNs; UUO, UUO surgery; UUO+Scr, underwent UUO surgery and were injected with scrambled ODNs; and UUO+mTOR/STAT, underwent UUO surgery and were injected with mTOR/STAT3 synthetic ODNs. (**C**) The graph of western blot results of cleaved caspase-3, cleaved poly (ADP-ribose) polymerase-1 (PARP-1), p53, and GAPDH. * *p* < 0.05 vs. NC group. † *p* < 0.05 vs. mTOR/STAT group. ‡ *p* < 0.05 vs. UUO group.

Renal tubular cell apoptosis is a critical detrimental event that leads to chronic kidney injury in association with renal fibrosis [36]. TUNEL staining indicated that more tubular cells underwent apoptosis in response to UUO surgery (Figure 7A). Tubular apoptosis was significantly increased in all obstructed kidneys in the UUO and UUO+Scr groups when compared to intact kidneys. In contrast, increased TUNEL-positive cell deaths were significantly attenuated by mTOR/STAT3 ODN treatment. Considering that apoptosis was increased in the kidneys of mice with UUO, we determined the effect of mTOR/STAT3 ODN on renal apoptosis using Western blots for apoptosis-related proteins. As shown in Figure 7B,C, there were significant increases in renal cleaved caspase-3, cleaved PARP1, and p53 expression in the UUO and UUO+Scr mice. Interestingly, UUO+mTOR/STAT mice showed a significant decrease in apoptosis-related proteins when compared to UUO mice. These results suggested that during chronic kidney injury induced by obstruction, mTOR/STAT3 synthetic ODN limited pro-apoptotic protein activation and inhibited apoptosis in tubular cells.

## 3. Discussion

In 2016, the Nobel Committee awarded the Nobel prize for Physiology or Medicine to Yoshinori Ohsumi for his novel work in identifying the biological process of autophagy and its critical function [37]. Therefore, studies of autophagy for the pathogenesis of numerous diseases, including renal injury, have attracted great interest, and they may provide valuable insights into potential therapeutic opportunities. Autophagy is a self-degradation process through which cells remove misfolded proteins, defective organelles, and damaged DNA to maintain cellular homeostasis [38]. Previous studies have demonstrated that autophagy in the kidney is vital for normal homeostasis, and the downregulation of autophagy is associated with AKI [39,40]. Autophagy also clears other cellular components, such as cytokines, and serves as an important tool for regulating inflammation [41].

Autophagy is known to have renal protective effects on renal tubular cells during AKI [42]. In addition, autophagy helps damaged kidneys repair and regenerate [43]. Therefore, impaired autophagy in kidneys resulted in inflammation and interstitial fibrosis in CKD models [43]. Xu et al. [44] also indicated that autophagy defects can lead to the excessive deposition of ECM and renal fibrosis. These studies demonstrated that autophagy induction is considered an effective therapy for preventing renal fibrosis or CKD [43]. Further, Peng et al. [45] found that autophagy deficiency owing to the deletion of ATG5 in proximal tubular epithelial cells (TECs) resulted in dramatically increased leukocyte infiltration and proinflammatory cytokines expression in UUO kidneys. However, the interaction between autophagy in tubules and renal inflammation is not completely understood. Many previous studies demonstrated that autophagy serves a dual purpose. It may play a cytoprotective role in the body [46] or promote cell injury and the development of CKD [11]. Kim et al. [11] indicated that renal fibrosis is accompanied by the upregulation of autophagy, whereas another study suggested that the downregulation of autophagy occurs in diabetic nephropathy [47]. These differences may be associated with the disease duration and stage of renal disease; autophagic downregulation is mainly observed in the early stages of diabetes, and enhanced autophagy is often observed in the late stages of diabetes and is associated with diabetic kidney fibrosis [47]. In this study, renal apoptosis and fibrosis were inhibited by the control of autophagy expression.

To clarify the specific role of autophagy in renal interstitial fibrosis, it is critical to examine the role of autophagy in matrix deposition by stimulating or deleting autophagy in renal tubular cells. However, how autophagy influences cell death in renal injury and what fundamental molecular interactions occur in the dynamic change of renal fibrosis after injury remain unknown [48]. An increasing number of studies have demonstrated autophagy induction in various experimental models of renal injury [49,50,51]. Moreover, the inhibition of autophagy aggravated AKI, whereas the activation of autophagy showed protective effects, suggesting the renal protective role of autophagy in AKI disease [52]. The inhibition of autophagy by 3-methyladenine (3-MA) could protect tubular cells from EMT and prevent fibrogenesis [53]. In contrast, 3-MA could further increase the apoptosis of renal TECs after obstructive nephropathy and tubule interstitial fibrosis, suggesting that autophagy is a renal protective mechanism in UUO [11]. Therefore, the underlying mechanism of autophagy-related renoprotection and anti-fibrotic mechanism remains unclear.

In addition, in response to renal injury, Atg5 and Atg7 knockout mice displayed dramatically increased tissue damage and apoptosis [54,55]. Compared to control mice, autophagy-deficient mice exhibited increased tubular damage, loss of renal function, tubular cell apoptosis, mitochondrial damage, and accumulation of p62 in response to renal injury [54,55,56,57]. In addition, transgenic mice with the deletion of Beclin-1 or LC3 showed increased deposition of collagen I [20]. Mice subjected to the UUO model revealed increased collagen deposition accompanied by increased TGF-β1 expression in the obstructed kidney [19].

The previous study [58] demonstrated an elevation of activated autophagy biomarkers, including LC3, Atg3, Atg5, Atg7, Atg12, and Atg16. These biomarkers were upregulated in the renal tissue of UUO mice, suggesting that autophagic activation may be associated with renal tissue fibrosis in these mice. In this study, LC3 and Beclin-1 were used to detect the biological markers of autophagy expression. Immunological detection methods were conducted for chemical mediators, such as TNF-a, IL-1β, IL-6, NGAL, Kim-1, Mac-2, and CD4 to observe the change in UUO-induced renal injury. In this study, caspase 3, PARP 1, and p53 were used to detect apoptosis.

Microtubule-associated protein 1 LC3, which was used in this study, plays a key role in the formation of autophagosomes [59]. The presence of LC3 in autophagosomes and its transformation into the downward migration of LC3-II are markers of autophagy [60]. Tian et al. [61] reported that LC3-II expression was decreased after treatment with an autophagy inhibitor. Currently, most studies employ LC3 as an autophagic biomarker. Autophagy and apoptosis play a significant role in regulating cell homeostasis and survival [62,63]. Autophagy can regulate apoptosis following molecular interactions between the key proteins of these pathways, including members of the Bcl-2 family, autophagy proteins, and caspases [52]. Beclin-1 binds to Bcl-2 and inhibits autophagy under normal conditions [64]. In the UUO model, autophagy cooperates with the apoptotic machinery by acting upstream of apoptosis and converging with the apoptotic pathway [12,65]. Anti-apoptotic proteins such as Bcl-2 have been shown to inhibit autophagy by binding to Beclin-1 [66,67]. Renal fibrosis and apoptosis are inhibited by the regulation of autophagy after the suppression of mTOR and STAT3 expression.

mTOR is a serine/threonine protein kinase in the PI3K-related kinase family that controls cellular growth, survival, and metabolism. Activated mTOR regulates mRNA translation, ribosomal biosynthesis, and protein translation [68,69], all of which reduce autophagy. Elevated mTOR levels were found to be accompanied by interstitial fibrosis and acute cellular rejection in transplant kidney biopsies [70]. STAT3 is a member of the STAT protein family [71]; it was identified as a transcription factor, and it participates in inflammation, tumorigenesis, and metabolic disorders [72,73,74]. STAT3 activates or inhibits autophagy in various cell types and in different environments [75,76].

A previous study showed that cells treated with STAT3 inhibitors can enhance autophagy [77,78,79]. Yokoyama et al. [80] also showed that the inhibition of p-STAT3 can induce autophagy. By contrast, Yang et al. [16] showed that the inhibition of STAT3 expression by drug or gene silencing reduced autophagic activity, as reflected by the decrease in LC3 expression, increase in p62 expression, and decrease in the number of autophagosomes. In this study, apoptotic numbers were remarkably decreased, and fibrosis was inhibited as STAT3 expression was suppressed. Although the relationship with mTOR/STAT and autophagy requires further study, autophagy numbers might have been suppressed by the STAT3 function in this study.

In LC3 knockout mice, autophagy has been reported to have a protective function for renal TIF through mature TGF-β1 degradation in renal TECs [19]. TGF-β1 play a central role in the pathogenesis of tissue fibrosis, and the overexpression of this protein in renal TECs resulted in widespread peritubular fibrosis and induction of autophagy [81]. Furthermore, cell culture studies indicated that TGF-β1 may activate autophagy in tubular cells [82]. Therefore, it seems that TGF-β1 may be controlled by autophagy, and in turn, this may regulate several critical aspects of kidney fibrosis. In the UUO-induced renal fibrosis model used in this study, autophagy induction protected fibrosis through the regulation of the expression of TGF-β1 and IL-1β. Therefore, autophagy could undoubtedly be a useful target for developing new protective treatments for CKD.

To improve a new therapeutic approach, synthetic ODN was used to suppress the expression of both mTOR and STAT3 using a combination of antisense ODN for mTOR and decoy ODN for transcription factor STAT3 [83]. Antisense ODN designed complementary nucleic acid fragments that specifically trigger through the selective ribonuclease H cleavage of the target mRNA in the nucleus [84]. The decoy ODN technique is employed to block the transcription factor through the use of a synthetic ODN containing consensus sequences of DNA binding sites, which works at the transcriptional level [31,85].

In summary, this study demonstrated the critical role of mTOR/STAT3-regulated autophagy in UUO-induced TIF of the kidney, and it may provide a theoretical basis for anti-fibrotic treatment in clinical practice. This study proved that the inhibition of mTOR and STAT3 expression has a therapeutic effect on preventing renal fibrosis. This study also showed that the regulation of autophagy can remarkably inhibit renal fibrosis through the downregulation of apoptosis. The combination of two chemical mediators, mTOR and STAT3, for the regulation of autophagy plays a beneficial role in terms of both preventative and therapeutic effects on renal injury. These results indicate that autophagy regulation by mTOR/STAT3 ODN administration in UUO-induced renal injury plays a protective role in TIF development and apoptosis through the regulation of the mTOR/STAT3 signaling pathway. Thus, autophagy in renal injury may represent a new therapeutic target for preventing renal TIF.

## 4. Materials and Methods

### 4.1. Construction of Synthetic ODNs

The target sites for mTOR were selected via the sequential overlap simulation of secondary structures using the S-Fold program. Synthetic ODNs were synthesized on a Macrogen. Synthetic ODN sequences were used as follows (target site of consensus binding sequence is underlined): scrambled (Scr) ODN: 5′-GAATTCAATTCAGGGTACGGCAAAAAATTGCCGTACCCTGAATT-3′; mTOR ODN: 5′-GAATTCCCCGAGUUCACACACGUCAAGGACGGG-3′; and STAT3 ODN (consensus sequence is underlined): 5′-GAATTCCCTTCCCGGAATTAAAAAATTCCGGGAAGG-3′. Scr ODN, mTOR ODN, and STAT3 decoy ODN were annealed for 6 h, while the temperature was decreased from 80 to 25 °C. To obtain a covalent ligation for ring-type ODN, each ODN was mixed with T4 ligase (Takara Bio Inc., Kusatsu, Japan) and incubated for 18 h at 16 °C.

### 4.2. Animal Model and Transfection of ODN

Male C57BL/6 mice (6 weeks old, 20–22 g; Samtako, Daejeon, South Korea) were housed individually in cages and maintained at a set temperature (22 ± 2 °C) and humidity (55%) with a 12 h light–dark cycle. After 1 week of acclimatization, the mice were randomly divided into five groups (*n* = 7 per group) as follows: (1) an untreated group (normal control, NC); (2) injected with the mTOR/STAT3 synthetic ODNs group (mTOR/STAT); (3) UUO surgery group (UUO); (4) underwent UUO surgery and were injected with the scrambled ODNs group (UUO+Scr); (5) underwent UUO surgery and were injected with the mTOR/STAT3 synthetic ODNs group (UUO+mTOR/STAT). For UUO surgery, each mouse was anesthetized, its abdominal cavity was incised, and the left ureter was ligated with 5–0 silk suture at both the distal and the proximal locations. The synthetic ODNs (10 μg) were injected into the mice intravenously at 2 days before ureteral ligation and 2 and 5 days after the UUO surgery. One week after the UUO operation, the mice were sacrificed. Figure 1B shows the design of the animal experiment. The animal protocols were approved by the Institutional Animal Care and Use Committee of the Catholic University of Daegu (EXP-IRB number: DCIAFCR-190620-07-Y).

### 4.3. Creatinine and Blood Urea Nitrogen

Mouse blood was obtained from the heart in all groups. After collection of the whole bloods, the bloods were allowed to clot by leaving them at room temperature (RT) for 2 h. The clots were removed by centrifugation at 2000 g for 20 min at RT. Sera were obtained from the supernatants after centrifugation. The samples were stored at −70 °C until analysis. Serum blood urea nitrogen (BUN) was measured using a BUN-E kit (Asan Pharm, Seoul, Korea). The analysis of samples was carried out according to the manufacturer’s recommended protocols. After mixing the BUN-E kit reagents and samples, they were reacted at RT for 15 min. The absorbance at 570 nm was determined using a microplate reader. Serum creatinine was measured using the QuantiChrom creatinine assay kit (Bioassay Systems, Hayward, CA, USA). We prepared the sample, standard, and working reagent according to the manufacturer’s recommended protocols. The diluted standard and serum were deposited into the wells of a clear-bottom 96-well plate. Next, we added the working reagent quickly to all wells. After 5 min, the absorbance at 510 nm was determined using a microplate reader.

### 4.4. Histological Analysis

All kidney tissue specimens were fixed in 10% formalin for 24 h at RT. After fixation, perpendicular sections to the anterior–posterior axis of the tissue were dehydrated in graded ethanol, cleared in xylene, and embedded in paraffin. The paraffin-embedded tissues were cut into 4 μm sections for deparaffinization. Kidney tissue sections were stained with hematoxylin and eosin (H&E), Masson’s trichrome, and periodic acid Schiff (PAS) according to the standard protocol. As part of the histological assessment, all slides were examined under a slide scanner (3DHISTECH Pannoramic MIDI, Budapest, Hungary).

### 4.5. Immunohistochemical (IHC) Staining

Paraffin-embedded tissue sections of 4 μm thicknesses were deparaffinized with xylene, dehydrated in gradually diminishing concentrations of ethanol, and treated with 3% hydrogen peroxidase in methanol for 10 min to block endogenous peroxidase activity. The tissue sections were immersed in a 10 mM sodium citrate buffer (pH 6.0) for 5 min at 95 °C. The last step was repeated using a 10 mM sodium citrate solution (pH 6.0). The sections were allowed to stay in the same solution while cooling for 20 min, following which they were rinsed in PBS. Then, the sections were incubated with a primary antibody (1:100 dilution) for 1 h at 37 °C. The primary antibody was as follows: anti-neutrophil gelatinase-associated lipocalin (NGAL, Santa Cruz Biotechnology, Santa Cruz, CA, USA), anti-kidney injury molecule-1 (Kim-1, formerly called Tim-1, Abcam, Cambridge, MA, USA), anti-Mac-2 (formerly called Galectin-3, Abcam), and anti-CD4 (Abcam). The signal was visualized using an Envision System (DAKO, Carpinteria, CA, USA) for 30 min at 37 °C. 3,3′-Diaminobenzidine tetrahydrochloride (DAB) was used as the coloring reagent, and hematoxylin was used as the counter-stain. The slides were examined using a slide scanner (Pannoramic MIDI) and analyzed with iSolution DT software (IMTechnology, Vancouver, BC, Canada).

### 4.6. Terminal Deoxyuncleotidyl Transferase-Mediated Digoxigenin-Deoxyuridine Nick-End Labeling (TUNEL) Staining and Confocal Microsocpy

TUNEL is a commonly used method for the detection of DNA fragmentation resulting from apoptotic signaling cascades. Apoptosis was analyzed using an in situ cell death detection kit (Roche Diagnostics, Indianapolis, IN, USA). Briefly, kidney sections were deparaffinized in xylene, rehydrated in graded ethanol solutions, and permeabilized. After washing, a TUNEL reaction mixture was added to the sections, following which they were incubated for 1 h at 37 °C. Nuclei were visualized using 4′,6-diamidino-2-phenylindole (DAPI) staining. TUNEL-positive cells were counted in 10 randomly chosen fields in each kidney at 400× magnification. Then, the stained slides were viewed under a confocal microscope system (Nikon A1 microscope equipped with a digital camera, Nikon, Tokyo, Japan).

### 4.7. Western Blot Analysis

The kidney tissues were homogenized in a protein lysis buffer for 20 min on ice and centrifuged at 12,000 rpm for 20 min at 4 °C. The supernatant was collected and the protein concentration was measured by the Bradford protein assay. Sodium dodecyl sulfate polyacrylamide gel electrophoresis was carried out with 8–12% polyacrylamide gels at 100 V for 1 h. The resolved proteins were transferred from the gel onto a nitrocellulose membrane (Millipore, Billerica, MA, USA) and probed with anti-TNF-α (Abcam), anti-IL-1β (Santa Cruz), anti-IL-6(Abcam), anti-fibronectin (Abcam), anti-collagen I (Abcam), anti- α-SMA (Abcam), anti-Beclin-1 (Cell Signaling Technology, Beverly, MA, USA), anti-p62 (Cell Signaling Technology), anti-light chain 3 (LC3) (Cell Signaling Technology), anti-cleaved-caspase3 (Cell Signaling Technology), anti-cleaved-poly (ADP-ribose) polymerase1 (PARP1, Santa Cruz), anti-p53 (Santa Cruz), and anti-glyceraldehyde 3-phosphate dehydrogenase (GAPDH, Cell Signaling Technology). This was followed by a secondary antibody conjugated to horseradish peroxidase (1:1000) and determined with enhanced chemiluminescence reagents (Amersham Biosciences, Piscataway, NJ, USA). The signal intensity was quantified by an image analyzer (Chemidoc XRS+ system; Bio-Rad Laboratories, Hercules, CA, USA).

### 4.8. Statistical Analysis

All data are presented as means ± standard error of the mean (SEM). Statistical significance was tested by one-way analysis of variance with Tukey’s multiple comparison test. Differences with *p* < 0.05 were considered significant.

## Data Availability

Data are contained within the article.

## Sample Availability

Samples of the compounds are not available from the authors.
